# Use of Herbal Dietary Supplement Si-Wu-Tang and Health-Related Quality of Life in Postpartum Women: A Population-Based Correlational Study

**DOI:** 10.1155/2013/790474

**Published:** 2013-02-14

**Authors:** Pei-Jen Chang, Ching-Chun Lin, Yi Chun Chen, Chao-Hua Chuang, Yu-Ching Tseng, Wu-Shiun Hsieh, Shio-Jean Lin, Pau-Chung Chen

**Affiliations:** ^1^Department of Nursing, National Taipei University of Nursing and Health Sciences, Taipei 112, Taiwan; ^2^Institute of Occupational Medicine and Industrial Hygiene, National Taiwan University College of Public Health, Taipei 100, Taiwan; ^3^School of Nutrition and Health Sciences, Taipei Medical University, Taipei 110, Taiwan; ^4^Department of Nursing, Chang Jung Christian University, Tainan 711, Taiwan; ^5^Department of Paediatrics, National Taiwan University Hospital and National Taiwan University College of Medicine, Taipei 100, Taiwan; ^6^Department of Paediatrics, Chi Mei Medical Center, Tainan 710, Taiwan; ^7^Department of Public Health, National Taiwan University College of Public Health, Taipei 100, Taiwan; ^8^Department of Environmental and Occupational Medicine, National Taiwan University Hospital and National Taiwan University College of Medicine, Taipei 100, Taiwan

## Abstract

*Objective*. The aim of the study was to explore the association between women's use of herbal dietary supplement Si-Wu-Tang during the postpartum period and their health-related quality of life. *Methods*. This is a population-based correlational study. We used multistage, stratified, systematic sampling to recruit 24,200 pairs of postpartum women and newborns from the Taiwan National Birth Registry in 2005. A structured questionnaire was successfully administered to 87.8% of the sampled population. Trained interviewers performed home interviews 6 months after the women's deliveries between June 2005 and July 2006. The Medical Outcomes Study 36-item Short-Form (SF-36) was used to measure the quality of life of the women along with the frequency of Si-Wu-Tang use. *Results*. Si-Wu-Tang use after delivery improved women's score for bodily pain and also improved their score for mental health when used more than 10 times. In addition, there were increases in general health and vitality scores in the group who continuously used Si-Wu-Tang more than 10 times after using Sheng-Hua-Tang. *Conclusion*. Use of Si-Wu-Tang after delivery may be associated with women's health-related quality of life especially for those who previously used Sheng-Hua-Tang. These results are exploratory and need to be replicated.

## 1. Introduction

Chinese postpartum care (zuo yuezi) has been regarded as a crucial rite-of-passage for the woman's recovery and the transition to motherhood after childbirth [1]. Previous studies have shown the beneficial effects of zuo yuezi on physical and mental health [2, 3] as well as social support [4]. During the postpartum period, the use of herbal dietary supplements is common and is frequently recommended by family members in Chinese communities [5]. The use of Sheng-Hua-Tang, which can relieve abdominal discomfort and eliminate lochia [6], is mostly common used during this period [7]. Our previous study has shown that Sheng-Hua-Tang use during the first month of the postpartum period may have a positive effect on women's health-related quality of life, especially in terms of role limitations due to physical health and emotional problems [8]. In Taiwan, Si-Wu-Tang, which is believed to speed the recovery from delivery, is commonly used after taking Sheng-Hua-Tang [7].

Si-Wu-Tang was originally listed in the Prescriptions from the Great Peace Imperial Grace Pharmacy and has been used as a basic formula in traditional Chinese medicine for the treatment of women's illnesses since the 12th century. The formula includes 4 ingredients: Radix Angelicae Sinensis 12.0 g, Rhizoma Ligustici Chuanxiong 6.0 g, Radix Rehmanniae Praeparata 9.0 g, and Radix Paeoniae Alba 9.0 g, and is available in many forms including pill, tablet, or capsule, drink or syrup, and natural or herbal form. Si-Wu-Tang is typically used for its action as a blood-tonifying (buxie) decoction [9] and is believed to stimulate the production of blood, promote blood circulation, regulate menstruation, and relieve menstrual pain [10]. A recent Cochrane review found promising evidence for the use of various Chinese herbal medicines in reducing menstrual pain in the treatment of primary dysmenorrhoea [11]. The majority of the 39 included trials used a complicated formula with more than 5 or 6 herbs. Two of them used modified Si-Wu-Tang and found that it resulted in a reduction of menstrual pain. Two recent studies also demonstrated that Si-Wu-Tang can decrease menstrual pain for primary dysmenorrhoea [12, 13].

Although there is evidence that supports the use of Si-Wu-Tang for primary dysmenorrhoea, its potential benefits in postpartum women have not been evaluated. It has been suggested to have four potential biological effects: anti-inflammation [14–18], uterine relaxation [19], haematological responses [20–24], and psychological responses [25, 26]. These effects improve postpartum women's overall physical and mental health.

Another interesting hypothesis needs to be examined. There may be additional beneficial effects of Si-Wu-Tang use in women with previous Sheng-Hua-Tang use in the postpartum period. Sheng-Hua-Tang includes 5 ingredients: Radix Angelicae Sinensis 24.0 g, Rhizoma Ligustici Chuanxiong 9.0 g, Semen Persicae 6.0–9.0 g, Zingiberis Rhizoma 1.5 g, and Glycyrrhizae Radix 1.5 g [6]. Both Si-Wu-Tang and Sheng-Hua-Tang share two common ingredients: Radix Angelicae Sinensis and Rhizoma Ligustici Chuanxiong. These two ingredients also have three similar biological effects: anti-inflammation [27], uterine relaxation [28], and haematological response [29–31]. These combined effects enhance the effects of Si-Wu-Tang on postpartum women's health-related quality of life.

It is common that Si-Wu-Tang is taken after Sheng-Hua-Tang use during the postpartum period to speed the recovery from delivery. Our previous study in Taiwan has shown that 44.8% of postpartum women used Si-Wu-Tang following Sheng-Hua-Tang use (82.6%) after delivery [7]. Although a high percentage of postpartum women use Si-Wu-Tang after the use of Sheng-Hua-Tang, no previous studies have investigated Si-Wu-Tang's potentially beneficial effects. The aim of the study was to explore the association between women's use of Si-Wu-Tang during the postpartum period and their health-related quality of life.

## 2. Materials and Methods

### 2.1. Study Design and Participants

A population-based correlational study was based on the Taiwan Birth Cohort Study (TBCS) which is the first national birth cohort study in Taiwan. In the current study, we used a multi-stage, stratified, systematic sampling design to obtain representative samples from the Taiwan National Birth registry in 2005. We ranked a total of 369 towns in Taiwan into 12 strata according to the administrative division (four strata) and the total fertility rate (three strata). Using the principle of proportion probability to size, we randomly sampled 90 towns out of 369 in Taiwan. A total of 24,200 pairs of postpartum women and newborns were recruited from these 90 towns [7].

We conducted home interviews of 24,200 women in the postpartum period, 6 months after their delivery, from June 2005 to July 2006. We lost 2,952 women to follow-up because of refusal to participate, changes in residence, incorrect addresses, infant deaths, and other reasons. A total of 21,248 postpartum women were interviewed. The completed interview rate was 87.8% [8]. We excluded incomplete questionnaires (*N* = 333), unmarried women (*N* = 586), handicapped or severely ill women (*N* = 160), women with pre-existing conditions, including diabetes mellitus, heart disease, and hypertension (*N* = 267) and postpartum depression (*N* = 139), women admitted to the hospital during the 6 months after childbirth (*N* = 269), and women with infants who had congenital defects or severe illnesses (*N*  =  1, 005). A total of 18,489 women were included in this study.

### 2.2. Data Collection

All study participants provided informed consent approved by the Ethics Review Board of the National Taiwan College of Public Health. Before the home interview, researchers delivered a card to notify the participating women about the interview and invited them to participate in the survey. After the women agreed to participate, interviewers visited the families at their homes and asked them to give informed consent after explaining the details of the study.

Data were collected using a structured questionnaire. We requested information on age, education, family income, employment status, parity, method of delivery, breastfeeding, postpartum nursing centre, outpatient clinic visits in the past 4 weeks, primary nursery by mother, use of Si-Wu-Tang and Sheng-Hua-Tang, and health-related quality of life.

### 2.3. Use of Si-Wu-Tang

Information regarding the pattern of use of Si-Wu-Tang was obtained from the interview questionnaire [7]. The questions included were “Did you use Si-Wu-Tang during the postpartum period?”; “When did you use Si-Wu-Tang during the postpartum period?”; and “How many times did you use Si-Wu-Tang during the postpartum period?”. We classified the frequency of Si-Wu-Tang use into three categories: 0, 1–10, and >10 times. We asked similar questions regarding the use of Sheng-Hua-Tang.

### 2.4. Health-Related Quality of Life Measures

The Medical Outcome Study 36-item Short-Form Health Survey (SF-36) is a generic health-related quality of life questionnaire [32]. The Taiwanese version was translated, back translated, and judged for similar meaning, and it demonstrated good reliability and validity in a healthy adult sample [33, 34]. It is identical to the original Version 1 of SF-36 and contains 36 items, which are grouped into the following eight scales: physical functioning, role limitations due to physical health problems, bodily pain, general health perceptions, vitality, energy or fatigue, social functioning, role limitations due to emotional problems, and general mental health. The score on each scale ranges from 0 to 100 with a higher score indicating better health or functioning.

### 2.5. Covariates

We considered potential confounders for quality of life, including maternal age (<25, 25–29, 30–34, ≥35 years), education (junior high school and below, senior high school, and university and above), family income per month (<50,000, 50,000–69,999, 70,000–99,999, ≥100,000 new Taiwan dollars, NT$, 1  US$ ≈ 30.5  NT$  in  2008), employment status (yes or no), parity (1, 2, ≥3), method of delivery (normal spontaneous delivery, Caesarean section), breastfeeding (ever, never), use of postpartum nursing centre (yes or no), out-patient clinic visits during the past 4 weeks (yes or no), infant sex (male, female), low birth weight or preterm (yes or no), primary nursery by mother (no, daytime, evening or night, whole day), Sheng-Hua-Tang use (yes, no), and time periods of Sheng-Hua-Tang and Si-Wu-Tang use (no use, within first month only, within first month and later).

### 2.6. Data Analysis

A chi-square test was used to compare the characteristics of subjects among the three groups by frequency of Si-Wu-Tang use. We analysed the association between the frequency of use of Si-Wu-Tang and women's health-related quality of life scores by one-way ANOVA. To control for potential confounders, the differences of SF-36 scores among the groups were evaluated by analysis of covariance (ANCOVA) and a least significant difference (LSD) multiple comparison test. The statistical threshold for significance was set at 0.05. The statistical analysis was performed using SPSS for Windows, Release 16.0.

## 3. Results

### 3.1. Characteristics of Study Participants

The characteristics of study participants according to frequency of use of Si-Wu-Tang during the 6-month postpartum period are shown in Table 1. Of the 18,489 women, 10,184 (55.1%) never used Si-Wu-Tang, and 5,806 (31.4%) and 2,499 (13.5%) used Si-Wu-Tang 1–10 times and more than 10 times during the 6-month postpartum period, respectively. There were 7,354 (39.8%) and 7,964 (43.1%) women who used Si-Wu-Tang with or without previous use of Sheng-Hua-Tang, respectively. Compared with women who did not use Si-Wu-Tang, those who used it 1–10 times or more than 10 times were slightly younger and had slightly lower levels of education and family income per month ≥70,000  NT$. In addition, these women were more likely to be multiparous, had stayed at postpartum nursing centres, and had more visits to out-patient clinics during the past 4 weeks.

### 3.2. Crude SF-36 Scores by the Frequency of Si-Wu-Tang Use

Table 2 presents crude SF-36 scores by the frequency of Si-Wu-Tang use during the 6-month postpartum period. The scores for vitality (*F* = 3.70, *P* = 0.03) and mental health (*F* = 7.52, *P* = 0.001) significantly increased in the groups who used Si-Wu-Tang more than 10 times compared with those who did not use it or used it 1–10 times. However, the highest score for physical function (*F* = 4.87, *P* = 0.01) was present in the group that used Si-Wu-Tang 1–10 times, and the highest score for role-emotional (*F* = 9.10, *P* < 0.001) was present in the group that did not use Si-Wu-Tang.

### 3.3. Adjusted SF-36 Scores by the Frequency of Si-Wu-Tang Use Stratified by Previous Sheng-Hua-Tang Use

**Figure 1:**
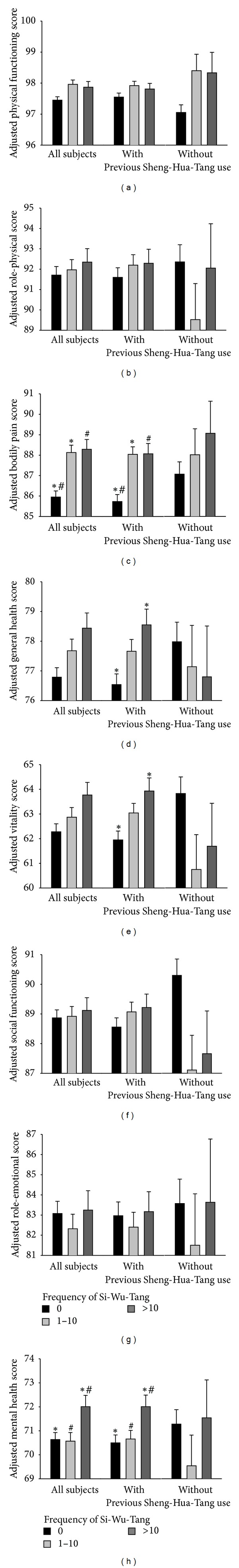
Adjusted SF-36 scores of study subjects according to the frequency of Si-Wu-Tang use during the 6-month postpartum period and stratified by previous Sheng-Hua-Tang use. Values were based on ANCOVA tests and adjusted for mother's age, education, family income per month, employment status, parity, method of delivery, breastfeeding, postpartum nursing centre, out-patient clinic visits during the past 4 weeks, infant low birth weight or preterm, primary nursery by mother, Sheng-Hua-Tang use, and duration of Sheng-Hua-Tang and Si-Wu-Tang use. ^∗,#^Scores differ significantly from each other according to a least significant difference (LSD) multiple comparison test. I bars indicate standard errors.

Figure 1 also shows adjusted SF-36 scores by the frequency of Si-Wu-Tang use during the 6-month postpartum period stratified by previous Sheng-Hua-Tang use. After controlling for potential confounders, the score for bodily pain (*F* = 6.54, *P* = 0.001) significantly improved in the groups who used Si-Wu-Tang either 1–10 times or more than 10 times compared with those who did not use Si-Wu-Tang. There was also a significant increase in the mental health score (*F* = 7.03, *P* = 0.001) in the group who used Si-Wu-Tang more than 10 times.

For women with previous Sheng-Hua-Tang use, the score for bodily pain (*F* = 6.27, *P* = 0.002) significantly improved in the groups who used Si-Wu-Tang either 1–10 times or more than 10 times compared with those who did not use Si-Wu-Tang. There were also significant increases in the general health (*F* = 3.50, *P* = 0.03), vitality (*F* = 3.44, *P* = 0.03), and mental health (*F* = 5.62, *P* = 0.004) scores in the group who used Si-Wu-Tang more than 10 times. However, no significant benefits were found in those without previous Sheng-Hua-Tang use.

## 4. Discussion

Our results indicate that Si-Wu-Tang use after delivery improved women's SF-36 score for bodily pain, and the use of Si-Wu-Tang more than 10 times during this period also increased their score of mental health. Additionally, the SF-36 scales displayed increases in the general health and vitality scores in the group who used Si-Wu-Tang more than 10 times after Sheng-Hua-Tang use. However, no significant benefits were found in women without previous Sheng-Hua-Tang use.

Si-Wu-Tang may benefit women's overall physical health, as shown in the bodily pain score of the SF-36 scale. It has been demonstrated to have pharmacological effects including the release of prostaglandin E2 [15], the reduction of cyclooxygenase-2 expression [18], anti-inflammatory activity [14, 15], the inhibition of histamine release [17], reducing coetaneous inflammatory diseases [16], and the inhibition of uterine contractions [19], which may be related to reduced physical pain. Si-Wu-Tang has also been shown to reduce blood-stagnation through the inhibition of platelet aggregation [21] and induce increased levels of circulating red blood cells, haemoglobin, and haematocrit [20, 22–24]. All of these biological effects might contribute to Si-Wu-Tang's effect on women's general health and well being during the postpartum period. Antimetastatic [35] and antitumour effects of Si-Wu-Tang against endometrial carcinoma have also been found and are likely related to the suppression of oestrogen receptor-alpha mRNA expression [36]. However, we do not know whether or not there is a direct link between these effects and postpartum women's health.

Si-Wu-Tang may also benefit women's mental health, as shown in the mental health score of the SF-36 scale. We do not know the mechanisms underlying this benefit. As previously mentioned, Si-Wu-Tang likely reduces menstrual pain and increases blood circulation and production. It may also improve women's overall physical health and, in turn, enhance their mental health status. Furthermore, paeoniflorin, one of the active components of Radix Paeoniae Alba, contributes to the cognitive-enhancing effects of Si-Wu-Tang [25, 26]. More research on other psychotropic effects of Si-Wu-Tang is warranted.

However, there was no clear dose-dependent relationship between Si-Wu-Tang and the improved score for bodily pain or mental health. Because we only asked about the frequency of Si-Wu-Tang use during the 6-month postpartum period, we do not know the exact dosage or the duration of use. In addition, the purposes for Si-Wu-Tang use vary and thus may require different dosages or durations. Dosage ratios among the 4 ingredients, additional ingredients, and preparation methods also vary for different decoction prescriptions. Thus, further randomised controlled trials are needed to answer the question.

Our previous study [8] found that the use of Sheng-Hua-Tang during the first month of the postpartum period significantly increased the SF-36 score in role limitations due to physical health and emotional problems but the use of Si-Wu-Tung in this study significantly improved the score in bodily pain and mental health. The effect of Sheng-Hua-Tang has been shown in animal models that it increases the myoelectric activity of rabbit uterine smooth muscle [37] and the contraction of the uterus when cotreated with oestrogen [38]. A recent study also showed that Sheng-Hau-Tang might increase the contractile activity and participate in the returning of the uterus to its anteverted position in postpartum women [39]. This might explain why the beneficial effects of Sheng-Hua-Tang are different from those of Si-Wu-Tang.

Additional benefits of Si-Wu-Tang use on general health and vitality based on the SF-36 scale were found in women with previous Sheng-Hua-Tang use in the postpartum period. This finding may result from the cumulative effects of their similar ingredients or their similar pharmacological effects. For example, both Sheng-Hua-Tang and Si-Wu-Tang share two common ingredients: Radix Angelicae Sinensis and Rhizoma Ligustici Chuanxiong which have three similar biological effects: anti-inflammation [27], uterine relaxation [28], and haematological response [29–31]. On the other hand, there were no significant benefits found in women with no previous Sheng-Hua-Tang use. Although there is no obvious explanation for this discrepancy, this implies that Si-Wu-Tang taken after Sheng-Hua-Tang use during the postpartum period may speed the recovery from delivery and return to prepregnancy state.

However, the differences between the groups were very small and may not be important in clinical significance. This may be partially due to the fact that our study is based on postpartum women from the general population rather than a specific population with menstrual disorders, such as primary dysmenorrhoea. Although we excluded women with major diseases, we do not know whether our population had any other menstrual symptoms or other reasons to use Si-Wu-Tang. Si-Wu-Tang can be obtained from any Chinese herbal medicine shop without a prescription by a doctor of Chinese medicine. Thus, we cannot generalise our results to any specific menstrual disorders.

These effects may be confounded by other aspects of Chinese postpartum care (zuoyuezi). Chinese postpartum care may provide valuable social support [4] and reduce physical and depressive symptoms [2, 3]. However, women whose caregiver was their mother-in-law or who perceived zuoyuezi as unhelpful had twice the odds for postpartum depression [40]. It has been found that conflict with the mother-in-law is a significant stressor for married Chinese women. If zuoyuezi is included in the model, there could be over- or under-adjustment of our estimates. Thus, we only included the variable regarding postpartum nursing centres after deliveries [41].

We have also considered other major potential confounders in our model, including the mother's age, education, family income per month, employment status, parity, method of delivery, breastfeeding, out-patient clinic visits during the past 4 weeks, infant low birth weight or preterm, and primary nursery by mother [42]. However, we did not explore every dimension that could potentially be related to women's quality of life. Although we attempted to collect all this information, we are concerned that the quality may be poor in this large cohort study.

Our study has potential limitations. The retrospective measurement of Si-Wu-Tang use may be subject to recall bias. We used a structured questionnaire administered by experienced interviewers to collect information. We first asked whether they used Si-Wu-Tang and the time period of use (during or after zuoyuezi) and then inquired about frequency [7]. The order of questions was meant to minimise recall bias. In addition, postpartum women usually obtain Si-Wu-Tang in finished herbal products from Chinese herbal stores rather than the whole herbs. The dosage ratios among the 4 ingredients, additional ingredients, and preparation methods may vary for decoction prescriptions. The possibility of random misclassification of the information exists, but this would not be expected to produce false-positive findings. We have also acknowledged that the cross-sectional study design may limit our conclusions. Nonetheless, the study results provide practical information for using Si-Wu-Tang in postpartum women.

## 5. Conclusions

Use of Si-Wu-Tang after delivery may be associated with women's health-related quality of life especially for those who previously used Sheng-Hua-Tang. Based on this and our previous studies [8], we suggested that Si-Wu-Tang taken after Sheng-Hua-Tang use during the first month of the postpartum period may benefit women's health. These results are exploratory and need to be replicated. Future studies employing a randomised, double-blind, placebo-controlled design are warranted.

## Figures and Tables

**Table 1:** Characteristics of study subjects according to the frequency of Si-Wu-Tang use during the 6-month postpartum period.

Characteristics	Frequency of Si-Wu-Tang use during				
	the 6-month postpartum period			χ^2^	*P*
	0	1–10	>10		
Total	10184	5806	2499		
Previous Sheng-Hua-Tang use (%)^a^					
Yes	78.2	88.7	88.2	343.35	<0.001
No	21.8	11.3	11.8		
*Mothers' characteristics *					
Age (%)^a^					
<25 years	18.9	20.3	19.8	44.87	<0.001
25–29 years	35.7	37.0	40.0		
30–34 years	32.1	31.6	29.8		
≥35 years	13.3	11.1	10.4		
Educational level (%)^a^					
Junior high school or below	15.1	13.3	13.3	43.44	<0.001
Senior high school	37.4	41.5	42.9		
University or above	47.5	45.2	43.8		
Employment status (%)^a^					
Yes	57.6	59.1	58.2	3.51	0.17
No	42.4	40.9	41.8		
Family income per month (%)^a^					
<50,000 NT$	40.8	42.2	40.7	19.94	0.003
50,000–69,999 NT$	25.5	27.1	26.9		
70,000–99,999 NT$	21.9	20.6	20.5		
≥100,000 NT$	11.8	10.1	11.9		
Parity (%)^a^					
1	51.7	48.4	48.6	28.46	<0.001
2	38.4	40.0	39.1		
≥3	9.9	11.6	12.3		
Method of delivery (%)^a^					
Normal spontaneous delivery	66.3	66.4	68.4	1.10	0.58
Caesarean section	33.7	33.6	32.6		
Breastfeeding (%)^a^					
Ever	82.6	82.7	83.7	1.70	0.43
Never	17.4	17.3	16.3		
Postpartum nursing centre (%)^a^					
Yes	6.9	7.5	8.5	8.44	0.02
No	93.1	92.5	91.5		
Out-patient clinic visits during the past 4 weeks (%)^a^					
Yes	31.1	33.2	31.9	7.47	0.02
No	68.9	66.8	68.1		
*Infants' characteristics *					
Sex (%)^a^					
Male	52.3	51.5	52.2	0.95	0.62
Female	47.7	48.5	47.8		
Low birth weight or preterm (%)^a^					
Yes	10.6	9.9	10.4	1.71	0.43
No	89.4	90.1	89.6		
Primary nursery by mother (%)^a^					
No	14.2	13.8	14.3	7.56	0.27
Daytime	2.1	2.1	2.8		
Evening or night	8.3	8.0	7.7		
Whole day	75.4	76.1	75.2		

^a^Calculated by column totals.

**Table 2:** Mean SF-36 scores and standard errors of study subjects according to frequency of Si-Wu-Tang use during the 6-month postpartum period.

SF-36 scales	Frequency of Si-Wu-Tang use during the 6-month postpartum period			*F*	*P*
	0	1–10	>10		
Physical functioning	97.54 ± 6.42*	97.85 ± 5.76*	97.74 ± 5.92	4.87	0.01
Role—physical	91.98 ± 22.42	91.66 ± 22.82	91.97 ± 22.64	0.39	0.68
Bodily pain	86.76 ± 16.53	87.17 ± 16.36	87.21 ± 16.31	1.54	0.22
General health	77.21 ± 17.44	77.15 ± 17.85	77.91 ± 17.16	1.83	0.16
Vitality	62.48 ± 17.53*	62.62 ± 17.15^#^	63.53 ± 17.48^∗,#^	3.70	0.03
Social functioning	88.93 ± 14.66	88.85 ± 14.69	89.06 ± 14.82	0.17	0.85
Role—emotional	83.76 ± 31.59^∗,#^	81.55 ± 33.54*	82.26 ± 32.51^#^	9.10	<0.001
Mental health	70.83 ± 15.83*	70.34 ± 15.96^#^	71.78 ± 15.76^∗,#^	7.25	0.001

^∗,#^Scores differ significantly from each other in the row according to a least significant difference (LSD) multiple comparison test.

## References

[1] Cheung NF, Mander R, Cheng L (2006). ‘Zuoyuezi’ after caesarean in China: an interview survey. *International Journal of Nursing Studies*.

[2] Heh SS, Coombes L, Bartlett H (2004). The association between depressive symptoms and social support in Taiwanese women during the month. *International Journal of Nursing Studies*.

[3] Chien LY, Tai CJ, Ko YL, Huang CH, Sheu SJ (2006). Adherence to “doing-the-month” practices is associated with fewer physical and depressive symptoms among postpartum women in Taiwan. *Research in Nursing and Health*.

[4] Heh SS, Fu YY, Chin YL (2001). Postpartum social support experience while “doing the month” in Taiwanese women. *Journal of Nursing Research*.

[5] Chen LL, Wang CC (2000). Attitude and behavior towards postpartum recuperation in traditional Chinese medicines. *Journal of Nursing Research (ROC)*.

[6] Fu QZ, Yang SZ, Liu DW (1999). *Fu Qing-zhus Gynecology*.

[7] Chuang CH, Chang PJ, Hsieh WS, Tsai YJ, Lin SJ, Chen PC (2009). Chinese herbal medicine use in Taiwan during pregnancy and the postpartum period: a population-based cohort study. *International Journal of Nursing Studies*.

[8] Chang PJ, Tseng YC, Chuang CH (2010). Use of Sheng-Hua-Tang and health-related quality of life in postpartum women: a population-based cohort study in Taiwan. *International Journal of Nursing Studies*.

[9] Liang QD, Lu XQ, Ma ZC (2004). Preliminary study on hematopoietic constituents of Si-Wu-Tang. *Zhongguo Zhong Yao Za Zhi*.

[10] Liu C, Tseng A, Yang S (2005). *Chinese Herbal Medicine: Modern Applications of Traditional Formulas*.

[11] Zhu X, Proctor M, Bensoussan A, Smith CA, Wu E (2008). Chinese herbal medicine for primary dysmenorrhoea. *Cochrane Database of Systematic Reviews*.

[12] Yeh LL, Liu JY, Lin KS (2007). A randomised placebo-controlled trial of a traditional Chinese herbal formula in the treatment of primary dysmenorrhoea. *PloS One*.

[13] Cheng JF, Lu ZYJ, Su YC, Chiang LC, Wang RY (2008). A traditional Chinese herbal medicine used to treat dysmenorrhoea among Taiwanese women. *Journal of Clinical Nursing*.

[14] Kojima S, Inaba K, Kobayashi S, Kimura M (1996). Inhibitory effects of traditional Chinese medicine Shimotsu-to and its included crude fractions on adjuvant-induced chronic inflammation of mice. *Biological and Pharmaceutical Bulletin*.

[15] Sakuma K, Ogawa M, Kimura M, Yamamoto K, Ogihara M (1998). Inhibitory effects of Shimotsu-to, a traditional Chinese herbal prescription, on ultraviolet radiation-induced cell damage and prostaglandin E2 release in cultured Swiss 3T3 cells. *Yakugaku Zasshi*.

[16] Tahara E, Satoh T, Toriizuka K (1999). Effect of Shimotsu-to (a Kampo medicine, Si-Wu-Tang) and its constituents on triphasic skin reaction in passively sensitized mice. *Journal of Ethnopharmacology*.

[17] Dai Y, But PPH, Chan YP, Matsuda H, Kubo M (2002). Antipruritic and antiinflammatory effects of aqueous extract from Si-Wu-Tang. *Biological and Pharmaceutical Bulletin*.

[18] Tagami K, Niwa K, Lian Z, Gao J, Mori H, Tamaya T (2004). Preventive effect of Juzen-taiho-to on endometrial carcinogenesis in mice is based on Shimotsu-to constituent. *Biological and Pharmaceutical Bulletin*.

[19] Xu FH, Uebaba K (1999). Effect of Kampo formulations (traditional Chinese medicine) on circulatory parameters. *Acupuncture and Electro-Therapeutics Research*.

[20] Ma ZC, Gao Y, Tang HL, Wang SQ (2003). The effects of si-wu-tang on serum protein of blood deficient mice induced by radiation. *Zhongguo Zhong Yao Za Zhi *.

[21] Yasuda T, Takasawa A, Nakazawa T, Ueda J, Ohsawa K (2003). Inhibitory effects of urinary metabolites on platelet aggregation after orally administering Shimotsu-To, a traditional Chinese medicine, to rats. *Journal of Pharmacy and Pharmacology*.

[22] Guo P, Ma ZC, Li YF, Liang QD, Wang JF, Wang SQ (2004). Effects of siwu tang on protein expression of bone marrow of blood deficiency mice induced by irradiation. *Zhongguo Zhong Yao Za Zhi*.

[23] Guo P, Liang QD, Hu JJ, Wang JF, Wang SQ (2005). The effect of Siwu Tang on Epo and G-CSF gene expression in bone marrow of irradiated blood deficiency mice. *Zhongguo Zhongyao Zazhi*.

[24] Liang QD, Gao Y, Tan HL (2006). Effects of four Si-Wu-Tang’s constituents and their combination on irradiated mice. *Biological and Pharmaceutical Bulletin*.

[25] Ohta H, Ni JW, Matsumoto K, Watanabe H, Shimizu M (1993). Peony and its major constituent paeoniflorin, improve radial maze performance impaired by scopolamine in rats. *Pharmacology Biochemistry and Behavior*.

[26] Watanabe H (1997). Candidates for cognitive enhancer extracted from medicinal plants: paeniflorin and tetramethylpyrazine. *Behavioural Brain Research*.

[27] Yang TH, Jia M, Mei QB (2006). Effect of Angelica sinensis polysaccharide fraction AP-3 on IL-2 and IFN-*γ* induction. *Acta Pharmaceutica Sinica *.

[28] Tuttle RS, Marmelstein L, Trad T, Reddy S, Radley T (1989). In vitro uterine response to tetramethylpyrazine, the active constituent of Chung Chong (a traditional Chinese medicine). *American Journal of Obstetrics and Gynecology*.

[29] Wang J, Xia XY, Peng RX, Chen X (2004). Activation of the immunologic function of rat Kupffer cells by the polysaccharides of Angelica sinensis. *Acta Pharmaceutica Sinica*.

[30] Li J, Wang YP (2005). Effect of angelica polysaccharide on bone marrow macrophage and its relationship to hematopoietic regulation. *Chinese Traditional and Herbal Drugs*.

[31] Liu JG, Xu FQ, Shi DZ, Dong GJ (2005). Effect of the extracts from Rhizoma Chuanxiong and Radix Paeouiae Rubra in different proportions on promoting blood circulation and removing blood stasis. *Traditional Chinese Drug Research and Clinical Pharmacology*.

[32] Huang IC, Wu AW, Frangakis C (2006). Do the SF-36 and WHOQOL-BREF measure the same constructs? Evidence from the taiwan population. *Quality of Life Research*.

[33] Lu JFR, Tseng HM, Tsai YJ (2003). Assessment of health-related quality of life in Taiwan (I): development and psychometric testing of SF-36 Taiwan version. *Taiwan Journal of Public Health*.

[34] Tseng HM, Lu JFR, Tsai YJ (2003). Assessment of health-related quality of life in Taiwan (II): norming and validation of SF-36 Taiwan version. *Taiwan Journal of Public Health*.

[35] Onishi Y, Yamaura T, Tauchi K (1998). Expression of the anti-metastatic effect induced by Juzen-taiho-to is based on the content of Shimotsu-to constituents. *Biological and Pharmaceutical Bulletin*.

[36] Lian Z, Niwa K, Onogi K, Mori H, Harrigan RC, Tamaya T (2006). Anti-tumor effects of herbal medicines on endometrial carcinomas via estrogen receptor-alpha-related mechanism. *Oncology Reports*.

[37] Hong M, Yu L, Ma C, Zhu Q (2003). Effect of extract from shenghua decoction on myoelectric activity of rabbit uterine muscle in the latest period of pregnancy. *Zhongguo Zhong Yao Za Zhi*.

[38] Zhao D, Zhan WH, Li LH, Nie FZ, Jiao JJ, Li Y (2006). Effects of different concentration extract from Shenghua decoction on contractile activity of the uterine smooth muscle isolated from normal, estrogen-treated and postpartum mice. *Zhongguo Zhong Yao Za Zhi *.

[39] Ho M, Li TC, Su SY (2011). The association between traditional Chinese dietary and herbal therapies and uterine involution in postpartum women. *Evidence-Based Complementary and Alternative Medicine*.

[40] Wan EY, Moyer CA, Harlow SD, Fan Z, Jie Y, Yang H (2009). Postpartum depression and traditional postpartum care in China: role of Zuoyuezi. *International Journal of Gynecology and Obstetrics*.

[41] Hung CH, Yu CY, Ou CC, Liang WW (2010). Taiwanese maternal health in the postpartum nursing centre. *Journal of Clinical Nursing*.

[42] Chen YC, Chie WC, Kuo SC, Lin YH, Lin SJ, Chen PC (2007). The association between infant feeding pattern and mother’s quality of life in Taiwan. *Quality of Life Research*.

